# Genetic association between rs1695 in glutathione S-transferase P1 and risk of periodontitis: a pilot study

**DOI:** 10.22099/mbrc.2023.46999.1815

**Published:** 2023

**Authors:** Rakshitha Vettri Saravanan, Anitha Pandi, Karthikeyan Murthykumar, Smiline Girija Aseervatham Selvi, Paramasivam Arumugam, Vijayashree Priyadharsini Jayaseelan

**Affiliations:** 1 Department of Microbiology, Saveetha Dental College and Hospitals, Saveetha Institute of Medical and Technical Sciences, Saveetha University, Chennai-77; 2 Centre for Cellular and Molecular Research, Saveetha Dental College and Hospitals, Saveetha Institute of Medical and Technical Sciences, Saveetha University, Chennai-77; 3 Department of Periodontics, Saveetha Dental College and Hospitals, Saveetha Institute of Medical and Technical Sciences, Saveetha University, Chennai-77

**Keywords:** Genotype, GSTP, Periodontitis, Polymorphism

## Abstract

The present study aims to determine the association between a genetic polymorphism of *GSTP1* (rs1695) and the risk of periodontitis. This study used a cross-sectional design and included subjects from the South Indian population. A total of 100 individuals enrolled at Saveetha Dental College and Hospital, Tamil Nadu were included in this study. The participants were divided into control (n=50) and periodontitis (n=50) based on clinical examination. Blood samples were collected. Genotyping was performed using specific primers spanning the polymorphic site. The genotypic frequencies for the rs1695 polymorphism were not significantly different between cases and controls.

## INTRODUCTION

The term "periodontitis" refers to a group of inflammatory conditions that affect the gums, periodontal ligament, and bone, which can lead to tooth loss and spread inflammation throughout the body. Although aggressive periodontitis can rarely affect adolescents, chronic periodontitis primarily affects adults [1]. In adult periodontitis, the inflamed pocket surface is thought to be a source of a variety of inflammatory mediators and cytokines that have implications for diabetes, atherosclerosis, and low birth weight in preterm infants [2]. Biological risk factors such as hypertension, high cholesterol, diabetes, genetic predisposition, and obesity, as well as behavioral risk factors such as poor diet, physical inactivity, and smoking, are common and preventable causes of this disease [3]. According to published studies, there are significant racial and ethnic differences in the prevalence and severity of the disease [4]. The underlying factors associated with the disease phenotype can be attributed to human genetic variations or polymorphisms. The polymorphisms in key genes could increase the risk of acquiring a disease and make an individual susceptible to the disorder.

In addition, periodontitis may be an important risk factor for the development of potentially malignant oral lesions that may progress to cancer if left untreated. Chronic inflammation characterized by the development of periodontitis may serve to modify the oral environment and promote the development of lesions [5]. In recent years, there has been an increase in the incidence of oral cancer among individuals without any habits [6]. In connection with this fact, the present study was designed to identify the association between rs1695 in *GSTP*1 gene and periodontitis in patients without any exposure to habits such as smoking or tobacco use. Several candidate gene polymorphisms have been evaluated in South Indian population with respect to chronic periodontitis [7, 8]. Since periodontitis is a complex disease associated with common variants whose cumulative effect contributes to the severity of the disease, it is imperative to analyze a panel of SNPs in a population to derive an association between the genetic marker and the disease phenotype. The rs1695 polymorphism is a missense variant with G as the ancestral and A as the variant allele. There are numerous reports discussing the association of this polymorphism with multifactorial diseases [9-12]. Despite the availability of such extensive data, the association between rs1695 and the risk of periodontitis in the South Indian population is very scarce.

## MATERIALS AND METHODS

This study used a cross-sectional design and included individuals from the South Indian population. A total of 100 age- and sex-matched subjects enrolled in the Saveetha Dental College and Hospital, Tamil Nadu, were recruited for this study. The participants were divided into control and periodontitis groups on the basis of clinical examination. The periodontitis group included 50 patients (34 male, 16 female) with a mean age of 39.0 ± 8.2 years. The control group included 50 healthy subjects (34 male, 16 female) with a mean age of 41.3 ± 7.5 years. A detailed history of dental treatment, family history of periodontitis, smoking habits, and general health concerns were obtained from the participants. Except for the presence of periodontitis, the patients included in this study were systemically healthy. Pregnant or lactating mothers, immunocompromised individuals, and participants who had undergone surgery within the previous 6 months were excluded from this study. The study was approved by the institutional ethics committee.

**Sample collection and DNA extraction: **A volume of 5 mL of venous blood was collected from the antecubital fossa and dispersed in a sterile tube containing a dash of ethylenediaminetetraacetic acid. It was mixed thoroughly to avoid clot formation. DNA isolation was performed according to the modified Miller protocol [13].

**Polymerase chain reaction and restriction endonuclease digestion: **The genotypes were assessed by polymerase chain reaction (PCR) amplification and restriction digestion. The primers forward 5’-GTAGTTTGCCCAAGGTCAAG-3’ and reverse 5’-AGCCACCTGAGG GGTAAG-3’ were used to amplify DNA spanning the G>A polymorphic site of the *GSTP1 *gene. The amplification of DNA was performed in 20‐μL volumes using 50 ng of genomic DNA, 5 pmol/μL each of the forward and reverse primers along with PCR Master Mix [Takara, Shiga, Japan]. The cycling conditions were as follows: initial denaturation at 94°C for 5 minutes, denaturation at 94°C for 35 seconds, annealing at 66°C for 30 seconds, extension at 72°C for 35 seconds, and a final extension at 72°C for 5 minutes. A 5‐μL volume of PCR product was checked on a 1% agarose gel, and 15 μL of PCR product was digested using BsmAI a restriction enzyme [New England Biolabs, Hitchin, UK]. Digestion was carried out at 37°C for 2 hours. The digested product was visualised on 2% agarose gel and the results were documented. A subset of amplicons representative of each genotype was subjected to bidirectional sequencing using BigDye terminator cycle sequencing kit and 3730XL Genetic analyzer. The sequence obtained was further analyzed using the [http://www.technelysium. com.au/chromas.html]. 

**Statistical analysis:** All statistical analyses were performed with SPSS version 23.0 for Windows [SPSS, Chicago, IL, USA]. The distribution of genotypes and allele frequencies in the chronic periodontitis and control groups were compared using the χ^2^ test. The risk associated with individual alleles or genotypes was calculated as odds ratio (OR) with 95% confidence interval (95% CI). Statistical significance was set at P<0.05 for all tests.

## RESULTS AND DISUUSION

The demographic details of the periodontitis patients and normal healthy subjects are shown in Table 1. The genotype frequencies of the *GSTP1* polymorphism among the periodontitis patients and normal healthy subjects are shown in Table 2. The study population was in good agreement with the Hardy-Weinberg equilibrium (χ^2^=2.77, df=1, P>0.05). Statistical analysis showed that there was no significant association between the rs1695 and the risk of periodontitis.

**Table 1:** Demographic details of periodontitis patients and normal healthy subjects

**Clinical Characteristics**	**Chronic Periodontitis group (n=50)**	**Control subjects (n=50)**
Male : Female	34:16	34:16
Mean age (years)	39.0 ± 8.2	41.3 ± 7.4
Clinical attachment loss (mm)	6.13 ± 1.29	0
Probing pocket depth (mm)	5.48 ± 1.15	1.60 ± 0.57
Gingival index (mm)	1.74 ± 0.22	0.76 ± 0.16

**Table 2:** Association between rs1695 and the risk of periodontitis

**Genotypes/** ** Alleles**	**Cases**	**Controls**	**OR**	**95% CI**	**P-value**
**AA**	27	25	1.0	-	-
**GA**	18	17	0.98	0.42-2.31	0.964
**GG**	5	8	0.58	0.17-2.01	0.388
					
**A**	72	67	1.0	-	-
**G**	28	33	0.79	0.43-1.44	0.443

The variants of the *GSTP1* have been associated with susceptibility to various diseases, including colon and lung cancer [14, 15]. In this context, the present study was designed to determine the prevalence of rs1695 among two groups of individuals, namely, normal subjects and patients with periodontitis from South Indian population to derive the association between the variants and the disease phenotype. Numerous studies have reported the association between oral cancer and periodontitis, a chronic inflammatory disease [16]. Camargo and team conducted experiments to demonstrate the association between chronic periodontitis and a polymorphism in DNA encoding GST enzymes that metabolize compounds derived from tobacco smoke. The study aimed to analyze the frequency of genotypes in polymorphisms located in genes, namely, *GSTM1*, *GSTT1*, *GSTP1* in a Mexican population. The data derived from the study showed a significant difference between the genotype frequencies of *GSTM1* gene polymorphism among smokers and non-smokers [17]. 

A study has shown that the AA genotype had a strong association with oral cancer patients exposed to the areca nut chewing habit of the northeastern Indian population. Furthermore, in silico analysis revealed that the AA genotype had a relatively weak detoxification ability to metabolize RAN (raw and unprocessed areca nut)/tobacco. Interestingly, in vitro experiments showed elevated levels of 8-oxo-2'-deoxyguanosine in AA genotype tumor samples [18]. Thus, chronic exposure to tobacco in combination with the *GSTP1* AA genotype increases the risk of developing oral cancer through accumulation of DNA lesions and lower phosphorylation of c-Jun, which controls apoptosis [19]. Since chronic periodontitis is often considered as one of the risk factors for oral cancer [20] and several other autoimmune diseases, the investigation of the polymorphic variants of the *GSTP1* happens to be imperative to determine the susceptibility of a population or group to debilitating diseases such as cancer.

Accumulating evidence has reported variations in periodontitis characteristics, most often attributed to microbial and host genetic factors [21]. The consequence of different combinations of alleles can lead to changes in tissue structure, inflammatory process and antibody responses. Therefore, the identification of genes that contribute to disease progression can be used to assess the risk status of patients with periodontitis. The allelic variations observed in the candidate genes may also influence the efficacy of therapy. Although there was no significant difference in genotype frequencies between the case and control groups, stratification of individuals based on reported habits and environmental exposures could help to gain more clarity on the influence of gene polymorphisms in relation to disease susceptibility. The present group of chronic periodontitis patients were non-smokers belonging to the same ethnic background. This is the first study of its kind that derives a comparison between normal healthy subjects and subjects with periodontitis without habits. Further studies will be directed towards the inclusion of smokers, individuals exposed to smokeless tobacco and related habits with chronic periodontitis.

## Conflict of Interest:

None.
